# *In Vivo* Antidiarrheal Potential of the Leaf Extract of *Maytenus addat* (Loes.) Sebsebe and Its Major Compound

**DOI:** 10.1155/2024/5922487

**Published:** 2024-05-20

**Authors:** Bonsa Mogose, Daniel Bisrat, Kaleab Asres

**Affiliations:** ^1^Department of Pharmaceutical Chemistry and Pharmacognosy, School of Pharmacy, College of Health Sciences, Addis Ababa University, P.O. Box 1176, Addis Ababa, Ethiopia; ^2^Department of Pharmacy, College of Health Sciences, Mizan-Tepi University, Tepi, Ethiopia

## Abstract

Diarrhea continues to be one of the top causes of death in children under the age of five, particularly in developing nations. In Ethiopian traditional medicine, a variety of medicinal plants are used to treat diarrhea. One of these plants is *Maytenus addat* (Loes.) Sebsebe (fam. Celastraceae), which is endemic to the Afromontane forests, especially along forest margins, of Ethiopia. The air-dried powdered leaves of *M. addat* were macerated with 80% methanol to yield a crude extract. Additionally, the powdered plant material underwent sequential solvent extraction using chloroform, methanol, and water to obtain solvent fractions. The 80% methanol leaf extract, solvent fractions, and an isolated compound from *M. addat* were evaluated for their antidiarrheal activity using castor oil-induced diarrheal model, anti-enteropooling test, and charcoal meal test in mice. The results showed that the 80% methanolic leaf extract significantly reduced the onset of diarrhea, the weight of feces, and the frequency of defecation in all the tested doses. The methanol and water fractions of the hydroalcoholic extract also exhibited dose-dependent antidiarrheal activity, with the methanol fraction showing the highest activity at 400 mg/kg dose. Subsequently, the most active methanol fraction was subjected to C-18 solid phase extraction, resulting in the isolation of a 3-hydroxyflavone, identified as quercetin by ESI-qToF-MS, ^1^H, and ^13^C-NMR spectroscopic techniques. Quercetin demonstrated a strong antidiarrheal activity in a dose-dependent manner. Thus, the present study provided evidence that the leaves of *M. addat* possess genuine antidiarrheal activity upholding the traditional medicinal use of the plant for the treatment of diarrhea. The findings also suggest that quercetin is responsible, in full or in part, for the activity of the plant.

## 1. Introduction

The problem of diarrhea is widespread globally, and it has high morbidity and mortality rates in developing countries, especially children under the age of 2 are particularly vulnerable due to limited access to clean water, sanitation conditions, and proper hygiene practices [1]. This combined with worsening nutritional and overall health conditions contributes to high morbidity and mortality rates in these countries [2]. Childhood diarrhea is a significant concern worldwide, with approximately 1.7 billion cases occurring each year. It is responsible for one in nine child deaths, resulting in a prevalence rate of 4.9 episodes per child per year. Shockingly, diarrhea is the second leading cause of death in children under the age of five [1]. It claims the lives of 525,000 children annually and greatly contributes to malnutrition in this age group [3]. Overall, diarrhea accounts for 21% of deaths in children under the age of five [4]. Particularly in Africa and South Asia, this illness is responsible for a staggering 82% of children under-five [5]. In Ethiopia, diarrhea has been identified as the cause of 20% of child mortality [6], and 12% of children under the age of five suffered from diarrhea in 2016 [7].

To address diarrhea, the United Nations International Children's Emergency Fund (UNICEF) and the WHO recommend the use of reduced osmolality oral rehydration solution (ORS) to reduce stool output and vomiting in patients with acute noncholera diarrhea [8]. Zinc supplementation is also advised as it has been shown to decrease the severity, duration, and recurrence of diarrhea within the following 2–3 months [9]. Antimotility drugs like loperamide, diphenoxylate, paregoric, and codeine can effectively reduce the frequency of diarrhea episodes in adults. However, due to potential side effects and the possibility of worsening disease progression in specific cases, these drugs are not recommended for infants or children [10].

Additionally, the rise of antibiotic-resistant bacterial strains causing diarrhea, such as multidrug-resistant *Shigella* spp. in Ethiopia, Mozambique, and Nigeria, further emphasized the need for alternative treatment options [11]. Medicinal plants have been explored as potential alternatives, with several plants including *Brucea antidysenterica* J. F. Miller, *Crotalaria incana* L., *Pycnostachys abyssinica* Fresen [12], *Calpurnia aurea* (Ait). Benth, *Gossypium barbadense* L. [13], and *Rubia cordifolia* L. being reported to have antidiarrheal properties [14].

*Maytenus addat* (Loes.) Sebsebe is a shrub or large tree which grows up to 30 m in height [15]. It is endemic to Ethiopia where it is known by various vernacular names such as Atata (Amharic), Shamene (Guragigna) [16], and Kombolcha (Afaan Oromoo) [17]. In Ethiopian traditional medicine, the aqueous leaf extract of *M. addat* has a history of being used to treat diarrheal diseases [17]. Therefore, this study aims to investigate the antidiarrheal activity of the active constituent(s) from *M. addat* leaves, known for their reputation in managing diarrhea.

## 2. Materials and Methods

### 2.1. General

A mixture of methanol-water (2 : 1) was used as a solvent system for RP-TLC. Solid phase extraction utilized Isolute C-18 (EC) columns (10 g, IST, Hengoed, UK). NMR spectra were recorded on a Bruker Avance DMX 400 FT-NMR spectrometer operating at 400 MHz for ^1^H and 100 MHz for ^13^C at room temperature with tetramethylsilane (TMS) as an internal standard. Chemical shifts are reported in ppm, and coupling constants (*J*) in Hz. Mass spectrum of the isolated compound was generated using an AB SCIEX Triple TOF 5600 mass spectrometer (Concord, ON, Canada) equipped with ESI (electro spray ionization) in the negative-ion mode time of flight mass spectrometry (−ve mode ESI-qTOF-MS).

### 2.2. Plant Material

Leaves of *M. addat* were collected from the Gullele Botanic Garden, Addis Ababa, in November 2021. The plant was authenticated by Mr. Melalku Wondafrash at the National Herbarium, Department of Plant Biology and Biodiversity Management, College of Natural and Computational Sciences, Addis Ababa University (AAU), where a voucher specimen (Collection number: MA01) was deposited for future reference.

### 2.3. Preparation of Hydroalcoholic Extract

The leaves of *M. addat* were cleaned and air-dried under shade at room temperature. After drying they were powdered using a mortar and pestle. The powdered leaves (500 g) were then subjected to maceration with 80% methanol (2.5 L) at room temperature for three days, during which regular shaking and stirring were performed. After three days, the extract was filtered using Whatman No. 1 filter paper, and the marc was remacerated with the same volume of solvent for an additional three days. The filtrates were combined, and the methanol was removed under reduced pressure (below 40°C) using a Rota vapor (Heidolph, Germany). The remaining aqueous solution was dried in an oven at a temperature not exceeding 40°C. The dried extract was transferred in to an amber-colored vial, labeled, and kept in a refrigerator at 4°C until use.

### 2.4. Preparation of Solvent Fractions

The powdered plant material underwent successive solvent extraction. First, it was exhaustively extracted with chloroform, and the solvent was evaporated to dryness under reduced pressure. The marc was then allowed to dry in an open air and sequentially extracted with methanol and distilled water. The resulting extracts were dried in a vacuum oven at 40°C. The extracts were weighed, transferred into an amber-colored vails, labeled, and kept in a refrigerator at 4°C until use.

### 2.5. Isolation of Compound

The dried methanol fraction (1 g) was subjected to solid phase extraction (SPE) on Isolute C-18 (EC) columns (10 g, IST, Hengoed, UK). The elution process involved collecting 120 fractions, each with a volume of 10 ml, using MeOH-H_2_O gradients. The MeOH concentration was gradually increased during the elution process. The collected fractions were then grouped based on their RP-TLC profiles into 11 subfractions: [(F1–F20), MeOH-H_2_O (50 : 50)], [(F21–F30), MeOH-H_2_O (55 : 45)], [(F31–F40), MeOH-H_2_O (60 : 40)], [(F41–F50), MeOH-H_2_O (65 : 35)], [(F51–F60), MeOH-H_2_O (70 : 30)], [(F61–F70), MeOH-H_2_O (75 : 25)], [(F71–F80), MeOH-H_2_O (80 : 20], [(F81–F90), MeOH-H_2_O (85 : 15)] [(F91–F100), MeOH-H_2_O (90 : 10)], [(F101–F110), MeOH-H_2_O [(95 : 5), and [(F111–F120)], MeOH (100%)]. Of these, fractions 31–60 gave a single spot on a reversed phase TLC using different solvent systems. These fractions were combined to obtain a yellow solid substance, coded MA-3. MA-3 was transferred into an amber-colored vial and stored in a refrigerator at 4°C until further use.

MA-3: yellow solid substance; *R*_*f*_ = 0.51 on a RP-TLC using methanol-water (2 : 1) as a solvent system. HRESI–qTOF–MS (−ve mode, Figure S1): *m/z* = 301.0350 [M-H]^−^ exact calculated mass = *m/z* 301.0348 mu [M-H]^−^, indicating a molecular formula of C_15_H_10_O_7_. ^1^H-NMR (ppm, Figure S2): 6.73 (H-6, *d*, *J* = 2.0 Hz); 6.77 (H-8, *d*, *J* = 2.0 Hz); 7.39 (H-5′, *d*, *J* = 8.5 Hz); 8.12 (H-6′, *dd, J* = 8.5, 2.1 Hz); 8.62 (H-2′, *d*, *J* = 2.1 Hz); 13.33 (H-5, *s*). ^13^C-NMR (ppm, Figure S3): 94.63 (C-8); 99.56 (C-6); 104.79 (C-10); 116.99 (C-2′); 116.99 (C-5′); 121.38 (C-6′); 123.35 (C-1′); 138.24 (C-3); 147.44 (C-3′); 148.08 (C-2); 149.96 (C-4′); 157.80 (C-9); 162.78 (C-5); 165.86 (C-7); 177.63 (C-4). ^1^H and ^13^C-NMR spectral data match with those reported for quercetin [18].

### 2.6. Experimental Animals

Healthy Swiss albino mice weighing between 20 and 35 g and aged 6–8 weeks of either sex were used for the experiments. The mice were obtained from the animal center of the Department Pharmacology and Clinical Pharmacy, School of Pharmacy (SoP), Addis Ababa University (AAU). Prior to the experiment, the mice were housed in plastic cages at room temperature, maintained on a 12 h light/dark cycle, and provided with free access to pellet food and water. They were acclimatized for one week before the start of the experiment. The care and handling of the mice adhered to international guidelines for the use and maintenance of experimental animals [19] and were approved by the Institutional Review Board of the SoP, AAU (approval code: ERB/SOP/461/15/2023).

### 2.7. Acute Oral Toxicity Test

Acute oral toxicity tests of the extract and fractions were conducted following the Organization for Economic Cooperation and Development (OECD) guideline 425 [20]. First, one female mouse was administered with a 2000 mg/kg dose of *M. addat* hydroalcoholic extract or fractions and observed for any signs of toxicity within the first hour, and during the subsequent 24 hours. As the mouse survived, the same 2000 mg/kg dose of the extract or fractions was administered to four mice; thus, five mice were used for each test substance. Then, the mice were followed up for fourteen days.

### 2.8. *In Vivo* Antidiarrheal Activity

#### 2.8.1. Grouping and Dosing

Three antidiarrheal models in mice, namely, castor oil-induced diarrhea, charcoal meal testing for gastrointestinal motility, and castor oil-induced enteropooling, were used. For each model, seventy mice of either sex were randomly divided into fourteen groups, with each group consisting of five mice: group I (negative controls); group II (positive controls); and groups III, IV, and V (treatment groups). The negative control group received 10 ml/kg of distilled water, the standard positive group received 3 mg/kg of loperamide, and the treatment groups (III, IV, and V) received 100 mg/kg, 200 mg/kg, and 400 mg/kg of the crude extract or solvent fractions, respectively, orally.

#### 2.8.2. Castor Oil-Induced Diarrhea

The mice were fasted for 18 h with access to water only. Animals in each group received their respective doses of the test substances: vehicle, 10 ml/kg of distilled water for negative control; loperamide 3 mg/kg for positive control. Similarly, the treatment groups received their respective doses of the extract or fractions. One hour after the treatment, diarrhea was induced by orally administering 0.5 ml of castor oil to each mouse. The mice were placed in separate metabolic cages lined with a transparent paper. The paper was altered each of 1 h for 4 h of observation. The onset of diarrhea, quantity and weight of wet feces, and total quantity and mass of stool output were recorded at regular intervals during the experimental period. The percentages of stool production and wet feces of diarrheal inhibition (% inhibition of excretion) were calculated using the following formulas [21].

(1) $$\begin{array}{c} \text{Percent of fecal output}=\frac{\text{Mean weight of wet feces of treatment group}}{\text{Mean weight of wet feces of negative control group}}\times 100,\\ \text{Percent inhibition of defecation}=\frac{\text{Mean wet feces defecation of }\left(-\text{ve control}-\text{test}\right)}{\text{Mean wet feces defecation of negative control}}\times 100. \end{array}$$

#### 2.8.3. Castor Oil-Induced Enteropooling Test

The method of Oghenesuvwe et al. [22] was adopted with slight modifications to establish the intraluminal liquid collection. The mice were fasted for 18 h and treated with the respective substances as mentioned before. After 1 h of treatment, each animal received 0.5 ml of castor oil by the oral route. One hour later, the mice were sacrificed by cervical dislocation and both ends of the small intestine of each animal were tied with thread. The mass of the tied intestine was weighed before and after removing the gut contents, and the colonic content was drained into a graduated cylinder and the volume measured. The percentage of intestinal secretion and weight of intestinal contents reduction were calculated using the formulas given below [23].

(2) $$\begin{array}{c} \text{Percent inhibition by using MVSIC}=\frac{\text{MVICC}-\text{MVICT}}{\text{MVICC}}\times 100, \end{array}$$

where MVSIC is the mean volume of small intestinal content; MVICC is the mean volume of the intestinal content of the negative control group; and MVICT is the mean volume of the intestinal content of the test group.

(3) $$\begin{array}{c} \text{Percent inhibition by using MWSIC}=\frac{\text{MWICC}-\text{MWICT}}{\text{MWICC}}\times 100, \end{array}$$

where MWSIC is the mean weight of small intestinal content; MWICC is the mean weight of intestinal content of the control; and MWICT is the mean weight of intestine content of the test/standard drug group.

#### 2.8.4. Charcoal Meal (Gastrointestinal Motility) Test

In this model, the mice were fasted for 18 h. The negative and positive control groups received 10 ml/kg of distilled water and 3 mg/kg of loperamide, respectively. The treatment groups received their respective graded doses of the extract or fractions. After one hour, all the mice received a 0.5 ml of castor oil, followed by 1 ml of 5% activated charcoal suspension after another hour. The mice were then sacrificed by cervical dislocation after 30 min, and the small intestine was removed and placed lengthwise on a piece of paper. The length passed by the activated charcoal meal and the entire length of the intestine were determined to calculate the percentage of inhibition and peristaltic index as shown below [23].

(4) $$\begin{array}{c} \text{Percent inhibition}=\frac{\text{Intestinal transit by charcoal meal}\left(\text{control}-\text{treated}\right)}{\text{Intestinal transit by charcoal meal in the control group}}\times 100,\\ \text{Peristaltic index}\left(\text{PI}\right)=\frac{\text{Mean distance travelled by charcoal meal}}{\text{Mean length of small intestine }}\times 100. \end{array}$$

#### 2.8.5. *In Vivo* Antidiarrheal Index (ADI)

The ADI of the 80% methanol extract, solvent fractions, and standard drug loperamide was determined by combining three parameters from the above models using the formula presented below [24, 25].

(5) $$\begin{array}{c} \text{ADI}=\sqrt[{3}]{\text{Dfreq}\times \text{Pfreq}\times \text{Gmeq},} \end{array}$$

where Dfreq is the delay in excretion time as a percentage of vehicle treated group; Pfreq is the drop in purging frequency in the number of wet feces as a percentage of the vehicle treated group; and Gmeq is the gut meal travel decrease as a percentage of vehicle treated group.

### 2.9. Data Analysis

The data were analyzed using SPSS (Statistical Package for Social Science) software version 26. A one-way analysis of variance (ANOVA) was conducted, followed by Tukey post hoc multiple comparison tests to compare the test groups. The results were presented as mean ± standard error of the mean (SEM) and *p* < 0.05 was considered statistically significant.

## 3. Results

### 3.1. Yields

Maceration of 500 g of the dried leaves of *M. addat* with 80% methanol afforded 125 g dark green extract (25% (w/w)). A solid phase extraction of the active methanol fraction over C-18 column yielded 1.24 g flavonoid labeled as MA-3.

### 3.2. Structural Elucidation of the Isolated Compound

**Figure 1:**
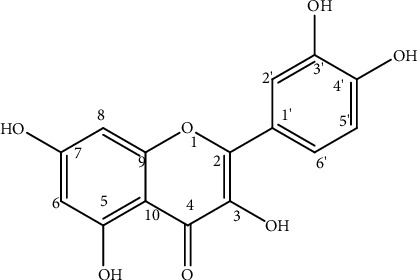
Chemical structure of quercetin.

MA-3 was isolated from the methanol fraction of *M. addat* leaves as a yellow solid substance. It gave an *R*_*f*_ value of 0.51 on a reversed-phase analytical TLC using methanol-water (2 : 1) as a solvent system. The negative-mode ESI-qTOF-mass spectrum of MA-3 (Figure S1) exhibited a pseudomolecular ion at *m/z* 301.0350 [M-H]^−^ (exact calculated mass = *m/z* 301.0348 mu [M-H]^−^), indicating a molecular formula of C_15_H_10_O_7_. The presence of 15 carbons was also evident from the ^13^C-NMR spectrum of MA-3, with one carbonyl carbon, seven oxygenated quaternary sp^2^ carbons, and two nonoxygenated sp^2^ quaternary carbons. This along with ^1^H-NMR spectrum as well as comparing its spectral data with previously reported data [19] for the same compound led to characterization of MA-3 as the 3-hydroxyflavone, quercetin (Figure 1).

### 3.3. Acute Oral Toxicity Study

None of the mice died during the acute oral toxicity study with the 80% methanol extract and solvent fractions of *M. addat* leaves, and no sign of toxicity was observed till the end of the 14^th^ day. Therefore, the LD_50_ of the plant is greater than 2000 mg/kg.

### 3.4. *In Vivo* Antidiarrheal Activity

#### 3.4.1. Effects on Castor Oil-Induced Diarrhea

Effects of the 80% methanol extract, solvent fractions, and quercetin isolated from the leaves of *M. addat* on castor oil-induced diarrhea are depicted in Table 1. The results indicate that all the test samples significantly decreased the frequency and quantity of castor oil-induced diarrhea, as well as the total weight of feces produced. The onset of diarrhea was also significantly (*p* < 0.05) delayed at all doses of the 80% methanol extract, with the highest effect observed at a dose of 400 mg/kg. Furthermore, the hydroalcoholic extract demonstrated dose-dependent inhibition of diarrhea frequency, with the 400 mg/kg dose showing the maximum effect. The positive control loperamide inhibited defection by 66.66% at a dose of 3 mg/kg, while inhibition caused by the 80% methanol extract at a dose of 400 mg/kg was 55.55%.

The solvent fractions also significantly (*p* < 0.05) delayed the onset of diarrhea compared to the negative control with the methanol fraction showing the highest activity (delaying the onset of diarrhea by 125.2 ± 1.43 min (*p* < 0.05) at a dose of 400 mg/kg). Both the aqueous and methanol fractions demonstrated dose-dependent reductions in the number of wet feces by 28.80%, 42.22%, and 60.00% for the aqueous fraction and 37.77%, 46.70%, and 62.20% for the methanol fraction at doses of 100, 200, and 400 mg/kg, respectively. However, the chloroform fraction showed a weaker effect compared to the other fractions.

The antidiarrheal activity of quercetin was also examined in this study, and the results showed that it significantly reduced the frequency and quantity of castor oil-induced diarrhea, as well as the total weight of feces produced. Quercetin also significantly (*p* < 0.05) delayed the onset of diarrhea in a dose-dependent manner. At a dose of 40 mg/kg, percent inhibition of defecation, percent inhibition of feces output, and the delay in the onset of diarrhea caused by quercetin were comparable to those of loperamide (Table 1).

#### 3.4.2. Effects on Castor Oil-Induced Enteropooling in Mice

The hydroalcoholic extract of *M. addat*, at all doses, significantly reduced intraluminal fluid accretion in mice compared to the vehicle-treated group (Table 2). The percentage volume of small intestinal content decreased by 32.9%, 60.5%, and 65.8% at doses of 100, 200, and 400 mg/kg, respectively. Also, the extract decreased the weight of the intestinal contents in a dose-dependent manner with the 400 mg/kg dose achieving a 58.2% reduction.

Both the aqueous and methanol fractions of the 80% methanol extract of *M. addat* significantly (*p* < 0.05) reduced the quantity of the intestinal contents compared to the vehicle-treated group (Table 2). The highest doses of these fractions resulted in the highest percentage reduction in both volume and weight of the intestinal contents, demonstrating 57.0% and 56.7% for the aqueous fraction and 68.4 and 60.2% for the methanol fraction, respectively. At a dose of 400 mg/kg, the activity of the methanol fraction in reducing both volume and weight of the small intestinal contents was comparable to that of loperamide (3 mg/kg). Again, the chloroform fraction did not show significant activity at any of the tested doses.

In the present study, quercetin was shown to reduce the volume and mass of intestinal content increased by the administration of castor oil. In mice, all evaluated doses of quercetin significantly reduced intraluminal fluid deposit compared to the vehicle-treated group (Table 2). The percentage volume of small intestinal content was reduced by 40.8%, 61.8%, and 71.1% at doses of 10, 20, and 40 mg/kg, respectively. Quercetin also dose-dependently decreased the weight of intestinal contents compared to the negative control, with the 40 mg/kg dose achieving a 61.22% reduction, similar to the positive control loperamide, which inhibited 61.2% at 3 mg/kg.

#### 3.4.3. Effects on Castor Oil-Induced Intestinal Transit in Mice

Table 3 provides data on the inhibitory effects of the 80% methanol extract of *M. addat* leaves, solvent fractions, and quercetin on the movement of a charcoal meal in the small intestine. The hydroalcoholic extract demonstrated percentage inhibition of 37.66, 45.71, and 52.52 on gastrointestinal transit time of charcoal meal at doses of 100, 200 and 400 mg/kg, respectively, in comparison to the control. On the other hand, the positive control, loperamide, displayed 66.76% (3 mg/kg) inhibition compared to the vehicle-treated group. Furthermore, the hydroalcoholic extract decreased movement of the charcoal meal by 46.48%, 40.70%, and 35.73% at doses of 100, 200 and 400 mg/kg, respectively, while the decrease caused by loperamide was 25.13%.

Similarly, the aqueous and methanol fractions exhibited dose-dependent inhibition of gastrointestinal motility. The aqueous fraction showed 44.23%, 47.95%, and 59.95% inhibition, while the methanol fraction displayed inhibition of 45.33%, 54.48%, and 64.33% at doses of 100, 200, and 400 mg/kg, respectively. In contrast, the chloroform fraction did not decrease intestinal motility (Table 3). The methanol fraction at a dose of 400 mg/kg significantly (*p*  <  0.01) suppressed movement of the charcoal meal within the small intestine and reduced the peristaltic index compared to the negative control. These effects were comparable to those observed for loperamide at a dose of 3 mg/kg. Thus, the plant extract inhibited gastrointestinal contractions and slowed down intestinal transits, allowing more time for water and electrolyte absorption.

The isolated compound quercetin also demonstrated antimotility activity, inhibiting the movement of charcoal meal in a dose-dependent manner. It showed percentage inhibition of 55.71%, 61.42%, and 66.66% at doses of 10, 20, and 40 mg/kg, respectively, while the positive control, loperamide, exhibited 66.76% (3 mg/kg) inhibition compared to the vehicle-treated group (Table 3). Additionally, quercetin reduced the mean percentage movement of the charcoal meal in the small intestine by 32.40%, 28.62%, and 24.91% at doses of 10, 20, and 40 mg/kg, respectively. At the highest dose employed, quercetin showed comparable activity with that of loperamide. Loperamide decreased movement of the charcoal meal in the small intestine by 25.13%.

#### 3.4.4. *In Vivo* Antidiarrheal Index

In this study, the total extract showed ADI values of 38.06, 57.79, and 70.91 at doses of 100, 200, and 400 mg/kg, respectively. Among the solvent fractions tested, the methanol fraction was the most effective demonstrating an ADI value of 79.42 at a dose of 400 mg/kg. On the other hand, the chloroform fraction had the lowest ADI value, indicating no significant antidiarrheal activity in all the parameters (Table 4).

At doses of 10, 20, and 40 mg/kg, quercetin demonstrated antidiarrheal effects with antidiarrheal index values of 68.03, 74.41, and 81.98, respectively.

## 4. Discussion

In Ethiopian traditional medicine, the crushed leaves of *M. addat* mixed with water are used for the treatment of diarrhea [18]. In this study, 80% methanol was chosen as an extracting solvent as it has been shown to be more effective in extracting both polar and nonpolar compounds from plant materials [26].

In the current study, the castor oil-induced diarrheal model was used to evaluate the antidiarrheal activity of *M. addat* leaves. This model was intended to evaluate the overall antidiarrheal activity of the test substances. Castor oil causes diarrhea through various ways. One of the mechanisms is that it produces the active metabolite ricinoleic acid by intestinal lipases. Ricinoleic acid stimulates the secretion of liquid and electrolytes and increases intestinal motility due to irritation and inflammation of the intestinal mucosa that leads to the release of prostaglandins [27, 28]. Castor oil also inhibits intestinal Na^+^/K^+^-ATP-ase activity, which induces diarrhea by reducing normal fluid absorption [29]. The other mechanism is activation of adenylate cyclase or mucosal cAMP-mediated active secretion as well as nitric oxide that induce diarrhea by hypersecretory response [27, 29]. Loperamide was used as a standard medication due to its antimotility and antisecretory properties, which effectively counteract the effects of castor oil [23]. Loperamide acts on the smooth muscle of the intestine similar to opiate antagonists, reducing propulsive motor activity primarily in the jejunum [30].

In the castor oil-induced diarrheal model, the 80% methanol extract of the leaves of *M. addat* significantly delayed the time of diarrheal onset and decreased the frequency of defecation and weight of feces in a dose-dependent manner. Therefore, this extract was further fractionated to determine whether the active components of the plant reside in the polar or nonpolar solvents. As shown in Table 1, the overall activity of the methanol and aqueous fractions was superior to those of the chloroform fraction suggesting that polar compound(s) could be responsible for the antidiarrheal activity of *M. addat* leaves. Moreover, in the castor oil-induced enteropooling test, the effect of the methanol fraction in reducing accumulation of fluid was significantly higher than the relatively nonpolar chloroform fraction, which was demonstrated by the drop of both volume and weight of intestinal contents (Table 2). In this model, the secreted fluid is accumulated in the lumen of the intestine as a result of inhibition of absorption caused by castor oil. Therefore, lesser volume of the intestinal content is an indication of antidiarrheal activity produced by a test substance. Similarly, in the castor oil-induced gastrointestinal motility model, the methanol fraction demonstrated a greater effect of reduction in the intestinal propulsive movement of charcoal meal than the aqueous and chloroform fractions (Table 3). The effectiveness of the methanol fraction in treating diarrhea was confirmed by the determination of its ADI. Unsurprisingly, the methanol fraction exhibited the highest ADI value compared to either the total extract or the other solvent fractions. As ADI is the combined effects of three diarrheal parameters, namely, purging frequency in the number of wet stools, delay in onset of diarrheal stool, and intestinal motility, a high value indicates a more successful treatment of diarrhea [27].

From the foregoing, it was deduced that the active antidiarrheal compounds reside mainly in the polar methanol fraction. Thus, phytochemical analysis of the methanol fraction was undertaken, which resulted in the isolation of quercetin as a major compound. In the present study, quercetin was shown to delay diarrheal onset and decrease stool frequency and weight of feces. It also showed dose-dependent decline in the weight and volume of intestinal contents and produced strong antimotility effect. More importantly, quercetin demonstrated a dose-dependent increase in *in vivo* antidiarrheal index. At a dose of 40 mg/kg, the ADI of quercetin (85.25%) was similar to the standard antidiarrheal agent loperamide (84.28%) revealing its effectiveness to cure diarrhea. Although quercetin was previously isolated from several plants including *Maytenus buchananii* [31], this is the first report on the isolation of quercetin from *M. addat*.

A previous study by Lozoya et al. [32] found that extracts of *Psidium guajava* leaves, traditionally used as herbal remedy for acute diarrhea in Mexico, inhibit peristalsis of guinea pig ileum *in vitro*. The active methanolic extract of the leaf extract was identified to be rich in flavanol glycosides, primarily based on quercetin. It was discovered that these glycosides are hydrolyzed by gastrointestinal fluid to active quercetin, which contributes to the antidiarrheal activity of *Psidium guajava*. Similarly, Zhang et al. [33] demonstrated that quercetin inhibits peristaltic motion in the mouse small intestine, decreases intestinal movement, and reduces capillary permeability in the abdominal cavity, offering a potential mechanism for the antidiarrheal effects of *P*. *guajava* extract. The present findings are also consistent with Carlo et al. [34] who reported that quercetin reduces intestinal transit and intraluminal accumulation of fluid in mice. Therefore, it is highly plausible that the antidiarrheal effect of *M. addat* is due to the presence of quercetin, which was previously shown to inhibit peristalsis by facilitating inhibitory enteric pathways [35]. Therefore, inhibition of the intestinal movement and reduction of capillary permeability in the abdominal activity could be the antidiarrheal mechanism of *M. addat* leaf extract.

## 5. Conclusions

The present study revealed that both the hydroalcoholic extract and solvent fractions of *M. addat* leaves possess *in vivo* antidiarrheal action in mice. Notably, the methanol fraction exhibited the strongest antidiarrheal effects. Phytochemical investigation of the methanol fraction led to the identification of 3-hydroxyflavone identified as quercetin. These findings provide scientific support for the traditional use of the *M. addat* leaves as an antidiarrheal medicine. The findings also furnished evidence that quercetin is responsible, in full or in part, for the antidiarrheal activity of the plant [36].

## Figures and Tables

**Table 1:** Effects of the 80% methanol leaf extract, solvent fractions, and quercetin isolated from *Maytenus addat* on castor oil-induced diarrhea.

Treatment (mg/kg)		Onset of diarrhea in min	Total # of wet feces output in 4 h	Percent inhibition of defecation	Total weight of wet feces (g) in 4 h	Percent inhibition of feces output	Total # of feces output in 4 h	Total weight of feces (g) in 4 h
NC 10 ml/kg		55.00 ± 7.90	9.00 ± 1.61	—	0.84 ± 0.07	—	12.80 ± 0.86	1.14 ± 0.09

Loperamide 3		129.00 ± 1.30^a2^	3.00 ± 0.45^a1^	66.66	0.34 ± 0.01	59.26	3.80 ± 0.49	0.42 ± 0.01

Extract	MA100	73.20 ± 2.90^ab1^	7.20 ± 0.86^b2^	20.00	0.68 ± 0.02	18.32	10.00 ± 1.23	0.81 ± 0.03
	MA200	105.60 ± 2.87^ab1^	5.40 ± 1.16	40.00	0.60 ± 0.03	28.14	7.40 ± 1.43	0.78 ± 0.03
	MA400	121.80 ± 2.65^abc1^	4.00 ± 0.84	55.55	0.49 ± 0.11^a2^	40.71	5.60 ± 0.93	0.67 ± 0.09

Fractions	CF100	56.20 ± 0.58	8.60 ± 0.60^b1e2^	4.44	0.71 ± 0.01^b1e2^	14.49	11.80 ± 0.66	0.96 ± 0.07
	CF200	57.80 ± 0.86	7.60 ± 0.68^b1^	15.55	0.69 ± 0.00^b2^	16.40	8.60 ± 0.75	0.74 ± 0.02
	CF400	68.00 ± 1.14^acd1^	6.40 ± 0.51	28.88	0.64 ± 0.02	23.11	7.40 ± 0.68	0.69 ± 0.01
	MF100	93.20 ± 1.24^a1b1j21^	5.60 ± 0.68	37.77	0.59 ± 0.02	28.62	7.00 ± 0.55	0.62 ± 0.02
	MF200	113.20 ± 1.07^a1b2i1^	4.80 ± 0.37^a2^	46.70	0.53 ± 0.01^a2^	37.01	5.00 ± 0.32	0.54 ± 0.01
	MF400	125.20 ± 1.43^a1i1j1^	3.40 ± 0.25^a2^	62.20	0.38 ± 0.02^a2^	54.01	4.20 ± 0.58	0.45 ± 0.03
	AF100	82.80 ± 1.77^a1b1^	6.40 ± 0.93	28.88	0.66 ± 0.01	20.71	8.60 ± 0.93	0.69 ± 0.01
	AF200	108.40 ± 0.92^a1b1i^	5.20 ± 0.80^a2^	42.22	0.54 ± 0.02^a2^	35.33	6.80 ± 0.74	0.58 ± 0.01
	AF400	122.60 ± 1.25^a1i1j2^	3.60 ± 0.68^a1^	60.00	0.48 ± 0.01^a1^	42.75	5.20 ± 0.37	0.49 ± 0.01

Quercetin	10	106.00 ± 0.70^ab^	4.20 ± 0.37^ab^	53.33	0.50 ± 0.00^ab^	40.48	5.20 ± 0.37^a^	0.54 ± 0.01^a^
	20	120.80 ± 0.73^abc^	4.00 ± 0.32^a^	55.53	0.45 ± 0.01^ab^	46.43	4.40 ± 0.24^a^	0.45 ± 0.01^a^
	40	128.40 ± 5.66^abcd^	3.20 ± 0.59^a^	64.44	0.35 ± 0.04^ac^	58.33	4.00 ± 0.79^a^	0.43 ± 0.06^a^

All values are articulated as mean ± standard error of the mean (SEM) (*n* = 5). Data were analyzed by one-way ANOVA followed by Tukey post hoc test. NC: negative control; MA100: *Maytenus addat* 80% methanol leaf extract (100 mg/kg); MA200: *Maytenus addat* 80% methanol leaf extract (200 mg/kg); MA400: *Maytenus addat* 80% methanol leaf extract (400 mg/kg); CF100: chloroform fraction (100 mg/kg); CF200: chloroform fraction (200 mg/kg); CF400: chloroform fraction (400 mg/kg); AF100: aqueous fraction (100 mg/kg); AF200: aqueous fraction (200 mg/kg); AF400: aqueous fraction (400 mg/kg); MF100: methanol fraction (100 mg/kg); MF200: methanol fraction (200 mg/kg); MF400: methanol fraction (400 mg/kg); a: compared to the control; b: compared to loperamide; c: compared to 100 mg/kg of MA; d: compared to 200 mg/kg of MA; e: compared to 400 mg/kg of MA; f: compared to 100 mg/kg CF; g: compared to 200 mg/kg of CF; h: compared to 400 mg/kg of CF; i: compared to 100 mg/kg of AF; j: compared 200 mg/kg of AF; k: compared to 400 mg/kg of AF; l: compared to 100 mg/kg of MF; m: compared to 200 mg/kg of MF; n: compared to 400 mg/kg MF; 1: *p*  <  0.01; 2: *p*  <  0.05; mice in the control group received distilled water (10 ml/kg).

**Table 2:** Effects of the 80% methanolic leaf extract, solvent fractions, and quercetin isolated from *Maytenus addat* on castor oil-induced enteropooling in mice.

Treatment	Dose (mg/kg)	MVSIC (ml)	Percent reduction MVSIC	MWSIC (g)	Percent reduction MWSIC
NC	10 ml/kg	0.76 ± 0.07	—	0.98 ± 0.15	—

Loperamide	3	0.25 ± 0.05^a1^	67.1	0.38 ± 0.03^a1^	61.2

Extract	MA100	0.51 ± 0.05^a1b1^	32.9	0.61 ± 0.09^a2^	37.9
	MA200	0.30 ± 0.05^a1^	60.5	0.45 ± 0.06^a1^	54.3
	MA400	0.26 ± 0.04^a1c1^	65.8	0.41 ± 0.02^a1^	58.2

Fractions	CF100	0.75 ± 0.01	1.3	0.97 ± 0.01	1.2
	CF200	0.71 ± 0.01	6.6	0.93 ± 0.01	5.1
	CF400	0.69 ± 0.01	8.4	0.90 ± 0.07	8.2
	AF100	0.52 ± 0.01^a1b1^	31.8	0.62 ± 0.01^a1^	36.9
	AF200	0.39 ± 0.01^a1^	48.4	0.47 ± 0.01^a1^	52.4
	AF400	0.33 ± 0.01^a1i1^	57.0	0.42 ± 0.01^a1^	56.7
	MF100	0.48 ± 0.01^a1b1k2^	36.8	0.58 ± 0.01^a1^	40.8
	MF200	0.35 ± 0.01^a1i2^	54.5	0.43 ± 0.02^a1^	56.1
	MF400	0.24 ± 0.02^a1i1j2l1^	68.4	0.39 ± 0.01^a1b1^	60.2

Quercetin	10	0.45 ± 0.01^a^	40.8	0.50 ± 0.01^a^	49.0
	20	0.29 ± 0.01^a^	61.8	0.39 ± 0.01^a^	60.2
	40	0.22 ± 0.01^ac^	71.1	0.38 ± 0.01^a^	61.2

All values are articulated as mean ± standard error of the mean (SEM) (*n* = 5). Data were analyzed by one-way ANOVA followed by Tukey post hoc test. NC: negative control; MVSIC: mean volume of small intestinal contents; MWSIC: mean weight of small intestinal contents; MA100: *Maytenus addat* 80% methanol leaf extract (100 mg/kg); MA200: *Maytenus addat* 80% methanol leaf extract (200 mg/kg); MA400: *Maytenus addat* 80% methanol leaf extract (400 mg/kg); CF100: chloroform fraction (100 mg/kg); CF200: chloroform fraction (200 mg/kg); CF400: chloroform fraction (400 mg/kg); AF100: aqueous fraction (100 mg/kg); AF200: aqueous fraction (200 mg/kg); AF400: aqueous fraction (400 mg/kg); MF100: methanol fraction (100 mg/kg); MF200: methanol fraction (200 mg/kg); MF400: methanol fraction (400 mg/kg); a: compared to the negative control; b: compared to loperamide; c: compared to 100 mg/kg of MA; d: compared to 200 mg/kg of MA; e: compared to 400 mg/kg of MA; f: compered to 100 mg/kg CF; g: compared to 200 mg/kg of CF; h: compared to 400 mg/kg of CF; i: compared to 100 mg/kg of AF; j: compared to 200 mg/kg of AF; k: compared to 400 mg/kg of AF; l: compared to 100 mg/kg of MF; m: compared to 200 mg/kg of MF; n: compared to 400 mg/kg of MF; 1: *p*  <  0.01; 2: *p*  <  0.05; mice in the control group received distilled water (10 ml/kg).

**Table 3:** Effect of 80% methanolic leaf extract, solvent fractions, and quercetin isolated from *Maytenus addat* on charcoal meal transit in castor oil-induced motility in mice.

Treatment	Dose (mg/kg) p.o	Mean length of small intestine (cm)	Mean distance travelled by charcoal meal (cm)	Percent charcoal meal transit (peristalsis index)	Percent inhibition
NC	10 ml/kg	56.24 ± 0.99	42.0 ± 0.97	74.70	—

Loperamide	3 mg/kg	55.54 ± 0.69	13.96 ± 0.75^a1^	25.13	66.76

Extract	MA100	56.32 ± 0.56	26.18 ± 0.38^a1b1^	46.48	37.66
	MA200	56.02 ± 1.23	22.80 ± 0.16^a1b1c1^	40.70	45.71
	MA400	55.8 ± 0.78	19.94 ± 0.51^a1b1c1d2^	35.73	52.52

Fractions	CF100	56.74 ± 0.43	39.50 ± 0.29^b1c1d1^	69.61	5.95
	CF200	56.70 ± 0.66	36.58 ± 0.57^a1b1c1d1^	64.51	13.09
	CF400	53.16 ± 0.60	34.70 ± 0.39^a1b1c1d1^	65.27	17.38
	AF100	54.36 ± 0.66	23.42 ± 0.47^a1b1^	43.08	44.23
	AF200	56.02 ± 0.58	21.86 ± 0.36^a1b1^	39.02	47.95
	AF400	53.44 ± 0.69	17.00 ± 0.35^a1b1i1j1^	31.81	59.52
	MF100	57.80 ± 0.86	22.96 ± 0.29^a1b1k1^	39.72	45.33
	MF200	52.46 ± 0.60	19.12 ± 0.28^a1b1i1j2l1^	36.44	54.48
	MF400	56.66 ± 0.41	14.98 ± 0.28^a1i1j1l1^	26.43	64.33

Quercetin	10	57.40 ± 0.51	18.60 ± 0.51^ab^	32.40	55.71
	20	56.60 ± 0.50	16.20 ± 0.64^a^	28.62	61.42
	40	57.00 ± 0.70	14.20 ± 0.25^ac^	24.91	66.66

All values are articulated as mean ± standard error of the mean (SEM) (*n* = 5). Data were analyzed by one-way ANOVA followed by Tukey post hoc test. p.o: peroral; NC: negative control; MA100: *Maytenus addat* 80% methanol leaf extract (100 mg/kg); MA200: *Maytenus addat* 80% methanol leaf extract (200 mg/kg); MA400: *Maytenus addat* 80% methanol leaf extract (400 mg/kg); CF100: chloroform fraction (100 mg/kg); CF200: chloroform fraction (200 mg/kg); CF400: chloroform fraction (400 mg/kg); AF100: aqueous fraction (100 mg/kg); AF200: aqueous fraction (200 mg/kg); AF400: aqueous fraction (400 mg/kg); MF100: methanol fraction (100 mg/kg); MF200: methanol fraction (200 mg/kg); MF400: methanol fraction (400 mg/kg); a: compared to the negative control; b: compared to loperamide; c: compared to 100 mg/kg of MA; d: compared to 200 mg/kg of MA; e: compared to 400 mg/kg of MA; f: compared to 100 mg/kg CF; g: compared to 200 mg/kg of CF; h: compared to 400 mg/kg of CF; i: compared to 100 mg/kg of AF; j: compared to 200 mg/kg of AF; k: compared to 400 mg/kg of AF; l: compared to 100 mg/kg of MF; m: compared to 200 mg/kg of MF; n: compared to 400 mg/kg MF; 1: *p*  <  0.01; 2: *p*  <  0.05; mice in the control group received distilled water (10 ml/kg).

**Table 4:** *In vivo* antidiarrheal index (ADI) of the 80% methanol extract, solvent fractions, and quercetin from *Maytenus addat*.

Treatment	Dose (mg/kg)	Dfreq (%)	Gmeq (%)	Pfreq (%)	*In vivo* ADI (%)
Extract	MA100	73.20	37.66	20.00	38.06
	MA200	105.60	45.71	40.00	57.79
	MA400	121.80	52.52	55.55	70.91

Fractions	CF100	56.20	5.95	4.44	11.40
	CF200	57.80	13.09	15.55	22.74
	CF400	68.00	17.38	28.88	32.43
	AF100	82.80	44.23	28.88	47.29
	AF200	108.40	47.95	42.22	60.31
	AF400	122.60	59.52	60.00	75.93
	MF100	93.20	45.33	37.80	54.25
	MF200	113.20	54.48	46.70	66.03
	MF400	125.20	64.33	62.20	79.42

Quercetin	10	106.00	55.71	53.33	68.03
	20	120.80	61.42	55.53	74.41
	40	128.40	66.60	64.44	81.98

Positive control	Loperamide 3 mg/kg	134.54	66.76	66.66	84.28

Dfreq: delay in the onset of diarrhea (min); Gmeq: gut meal movement reduction in intestinal transit; Pfreq: purging frequency in quantity of wet feces inhibition; MA100: *Maytenus addat* 80% methanol leaf extract (100 mg/kg); MA200: *Maytenus addat* 80% methanol leaf extract (200 mg/kg); MA400: *Maytenus addat* 80% methanol leaf extract (400 mg/kg); CF100: chloroform fraction (100 mg/kg); CF200: chloroform fraction (200 mg/kg); CF400: chloroform fraction (400 mg/kg); AF100: aqueous fraction (100 mg/kg); AF200: aqueous fraction (200 mg/kg); AF400: aqueous fraction (400 mg/kg); MF100: methanol fraction (100 mg/kg); MF200: methanol fraction (200 mg/kg); MF400: methanol fraction (400 mg/kg).

## Data Availability

The data associated with this study are included within the article. Additional files (NMR and ESI-MS spectra of quercetin) are available online as supplemental materials.

## Supplementary Materials

Supplementary Materials Figure S1: negative-mode electrospray ionization mass spectrum (−ve mode ESI-MS) of compound MA-3. Figure S2: ^1^H-NMR spectral data of MA-3. Figure S3: ^13^C-NMR spectral data of MA-3.
