# Supplementary material for: In Vivo Antidiarrheal Potential of the Leaf Extract of Maytenus addat (Loes.) Sebsebe and Its Major Compound
Source: J Trop Med. 2024 May 20;2024:5922487. doi: 10.1155/2024/5922487 (PMC11129909; doi:10.1155/2024/5922487)
Supplement: Supplementary Materials — Figure S1: negative-mode electrospray ionization mass spectrum (−ve mode ESI-MS) of compound MA-3. Figure S2: 1H-NMR spectral data of MA-3. Figure S3: 13C-NMR spectral data of MA-3. [file 5922487.f1.docx]

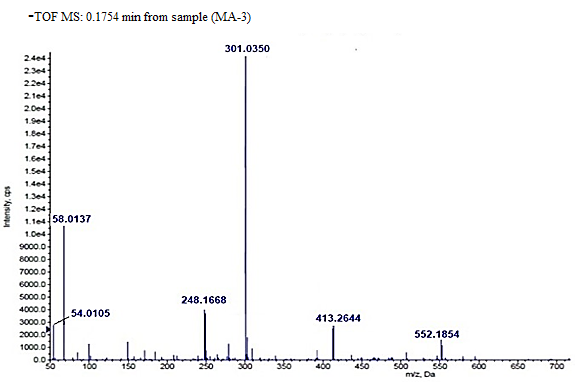


**Figure S1**: Negative-mode electrospray ionization mass spectrum (-ve mode ESI-MS) of compound MA-3


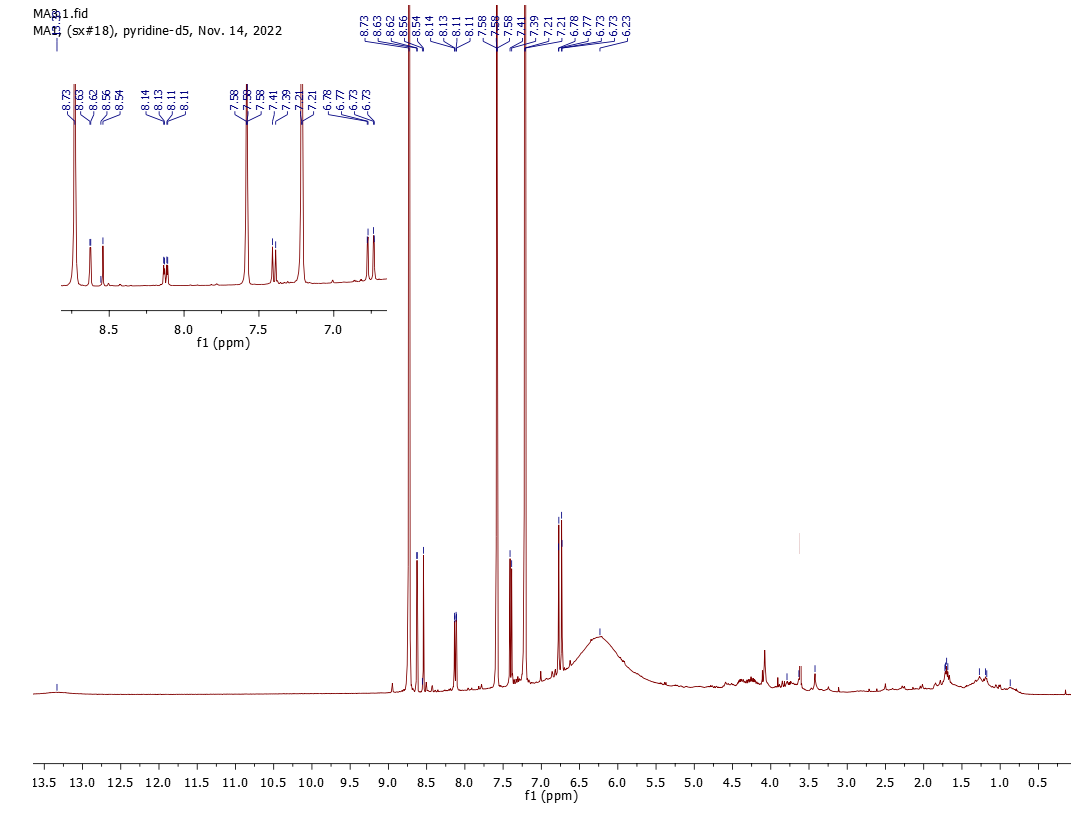


**Figure S2**: ^1^H-NMR spectral data of MA-3


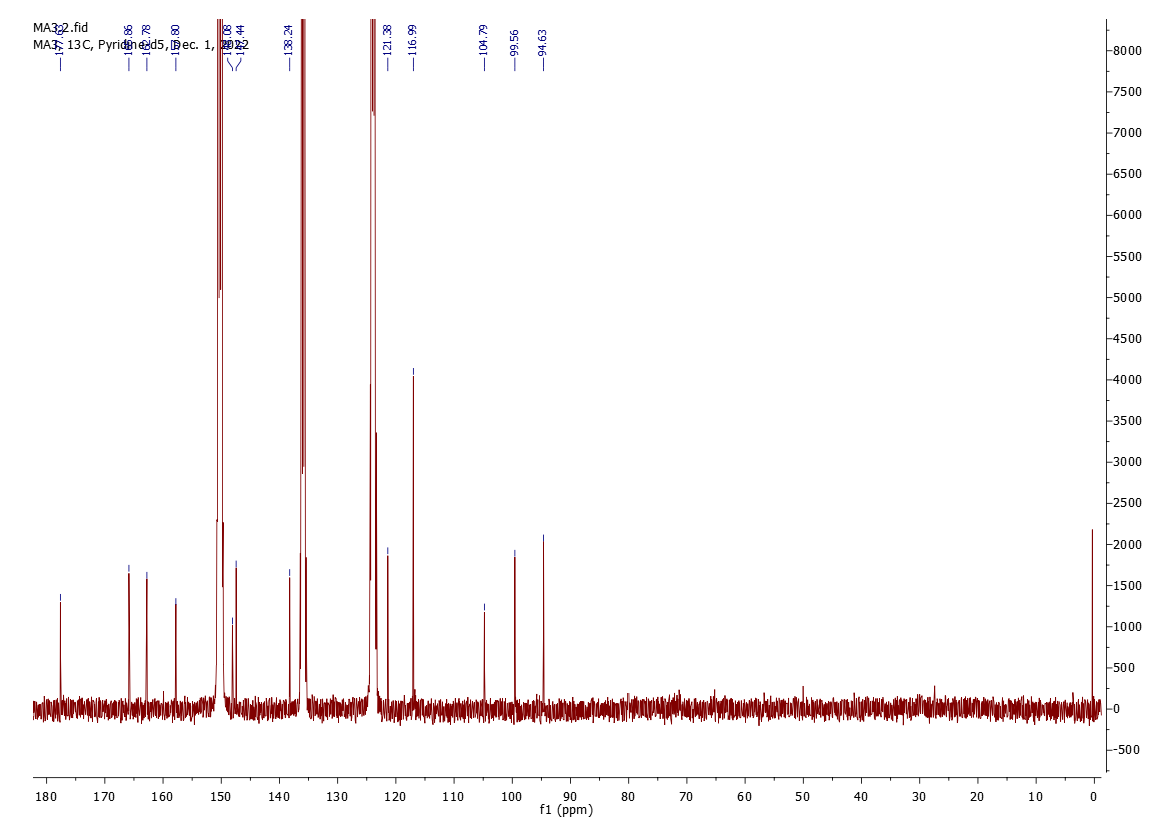


**Figure S3**: ^13^C-NMR spectral data of MA-3
